# The Use of SMS Text Messaging to Improve the Hospital-to-Community Transition in Patients With Acute Coronary Syndrome (Txt2Prevent): Results From a Pilot Randomized Controlled Trial

**DOI:** 10.2196/24530

**Published:** 2021-05-14

**Authors:** Emily S Ross, Brodie M Sakakibara, Martha H Mackay, David G T Whitehurst, Joel Singer, Mustafa Toma, Kitty K Corbett, Harriette G C Van Spall, Kimberly Rutherford, Bobby Gheorghiu, Jillianne Code, Scott A Lear

**Affiliations:** 1 Department of Biomedical Physiology and Kinesiology Simon Fraser University Burnaby, BC Canada; 2 Centre for Chronic Disease Prevention and Management University of British Columbia Okanagan Kelowna, BC Canada; 3 Department of Occupational Science and Occupational Therapy University of British Columbia Vancouver, BC Canada; 4 School of Nursing University of British Columbia Vancouver, BC Canada; 5 Centre for Health Evaluation and Outcome Sciences Vancouver, BC Canada; 6 Faculty of Health Sciences Simon Fraser University Burnaby, BC Canada; 7 Centre for Clinical Epidemiology and Evaluation Vancouver Coastal Health Research Institute Vancouver, BC Canada; 8 School of Population and Public Health University of British Columbia Vancouver, BC Canada; 9 Division of Cardiology Providence Health Care Vancouver, BC Canada; 10 School of Public Health Sciences University of Waterloo Waterloo, ON Canada; 11 Department of Medicine McMaster University Hamilton, ON Canada; 12 Department of Health Research Methods, Evidence and Impact McMaster University Hamilton, ON Canada; 13 Population Health Research Institute Hamilton, ON Canada; 14 Department of Family Practice University of British Columbia Vancouver, BC Canada; 15 Canada Health Infoway Toronto, ON Canada; 16 Department of Curriculum and Pedagogy University of British Columbia Vancouver, BC Canada; 17 Division of Cardiology Providence Health Care Healthy Heart Program St Paul's Hospital Vancouver, BC Canada

**Keywords:** SMS text messaging, mHealth, acute coronary syndrome, cardiovascular disease

## Abstract

**Background:**

Acute coronary syndrome (ACS) is a leading cause of hospital admission in North America. Many patients with ACS experience challenges after discharge that impact their clinical outcomes and psychosocial well-being. SMS text messaging has the potential to provide support to patients during this postdischarge period.

**Objective:**

This study pilot tested a 60-day SMS text messaging intervention (Txt2Prevent) for patients with ACS. The primary objective was to compare self-management domains between usual care and usual care plus Txt2Prevent. The secondary objectives were to compare medication adherence, health-related quality of life, self-efficacy, and health care resource use between groups. The third objective was to assess the feasibility of the study protocol and the acceptability of the intervention.

**Methods:**

This was a randomized controlled trial with blinding of outcome assessors. We recruited 76 patients with ACS from St. Paul’s Hospital in Vancouver, Canada, and randomized them to 1 of 2 groups within 7 days of discharge. The Txt2Prevent program included automated 1-way SMS text messages about follow-up care, self-management, and healthy living. Data were collected during the index admission and at 60 days after randomization. The primary outcome was measured with the Health Education Impact Questionnaire (heiQ). Other outcomes included the EQ-5D-5L, EQ-5D-5L Visual Analog Scale, a modified Sullivan Cardiac Self-Efficacy Scale, and Morisky Medication Adherence Scale scores, and self-reported health care resource use. Analyses of covariance were used to test the effect of group assignment on follow-up scores (controlling for baseline) and were considered exploratory in nature. Feasibility was assessed with descriptive characteristics of the study protocol. Acceptability was assessed with 2 survey questions and semistructured interviews.

**Results:**

There were no statistically significant differences between the groups for the heiQ domains (adjusted mean difference [Txt2Prevent minus usual care] for each domain—Health-directed activity: –0.13, 95% CI –0.39 to 0.13, *P*=.31; Positive and active engagement in life: 0.03, 95% CI –0.19 to 0.25, *P*=.76; Emotional distress: 0.04, 95% CI –0.22 to 0.29, *P*=.77; Self-monitoring and insight: –0.14, 95% CI –0.33 to 0.05, *P*=.15; Constructive attitudes and approaches: –0.10, 95% CI –0.36 to 0.17, *P*=.47; Skill technique and acquisition: 0.05, 95% CI –0.18 to 0.27, *P*=.69; Social integration and support: –0.12, 95% CI –0.34 to 0.10, *P*=.27; and Health services navigation: –0.05, 95% CI –0.29 to 0.19, *P*=.69). For the secondary outcomes, there were no statistically significant differences in adjusted analyses except in 1 self-efficacy domain (Total plus), where the Txt2Prevent group had lower scores (mean difference –0.36, 95% CI –0.66 to –0.50, *P*=.03). The study protocol was feasible, but recruitment took longer than expected. Over 90% (29/31 [94%]) of participants reported they were satisfied with the program.

**Conclusions:**

The Txt2Prevent study was feasible to implement; however, although exploratory, there were no differences between the 2 groups in adjusted analyses except for 1 self-efficacy domain. As the intervention appeared acceptable, there is potential in using SMS text messages in this context. The design of the intervention may need to be reconsidered to have more impact on outcome measures.

**Trial Registration:**

ClinicalTrials.gov NCT02336919; https://clinicaltrials.gov/ct2/show/NCT02336919

**International Registered Report Identifier (IRRID):**

RR2-10.2196/resprot.6968

## Introduction

Acute coronary syndrome (ACS), which includes unstable angina and myocardial infarction, is a leading cause of hospitalization in North America [1,2]. Once discharged, 20%-34% of patients are readmitted within 30 days [3,4]. While reducing readmissions is a complex issue, patients with ACS may experience several challenges after discharge that negatively impact their clinical outcomes and psychosocial well-being. One-third of patients with ACS do not adhere to the behavioral advice regarding diet, physical activity, and smoking cessation [5]. In Canada, 44% of patients with myocardial infarction do not have early physician follow-up within 7 days, particularly those who live in rural areas and lower-income neighborhoods [6]. Physician follow-up may be important to reduce readmissions and improve medication adherence [7-10]. Additionally, around a quarter of cardiac medication prescriptions are not filled within the first week of discharge [11]. Patients also report that once they return home they can feel overwhelmed or uncertain [12], be fearful of another cardiac event [13], and experience depression [12,14]. Their time in the hospital can be busy and overwhelming, and as such, some patients may have difficulty remembering everything they are told or may not know what to ask [12,15]. Furthermore, patients’ length of stay in the hospital has markedly declined over the past several decades [16,17], which means there can be less time to deliver patient education. Therefore, providing continuing support after hospital discharge may affect several key factors of post-ACS management, including lifestyle changes, medication adherence, and psychosocial well-being.

Home-based programs, often nurse led, can improve quality of life and reduce readmissions [18,19], but these face-to-face interventions can be a challenge for strained health care systems. The widespread use of information and communication technology, such as mobile phones, may be an easier and more convenient way to reach patients. SMS text messages are an attractive technology, as over 90% of adults aged 65 years or older own a cell phone [20], and 80% of cell phone owners currently text [21]. SMS text messages also have the benefits of being able to store messages that can be reaccessed, have a wide geographic reach, are convenient due to the asynchronous nature of communication, and have low delivery costs. Previous SMS text messaging studies in patients with or at risk for cardiovascular disease (CVD) have reported improvements in self-management behaviors (eg, medication adherence [22] and increases in leisure physical activity and walking [23]) and cardiac risk factors (eg, lowering low-density lipoprotein cholesterol and systolic blood pressure [24,25]). These studies show the promise of using SMS text messaging to aid in the care of patients with CVD. However, they do not specifically target the multiple self-management behaviors required in the immediate period after discharge using only SMS text messages. We report on a pilot study of a one-way SMS text messaging intervention (Txt2Prevent) aimed at supporting patients with ACS after hospital discharge in an assessor-blinded randomized controlled trial. The study’s primary objective was to assess the effect of the Txt2Prevent intervention on self-management domains compared with usual care. The other objectives were to compare quality of life, self-efficacy, medication adherence, and health care resource use between the 2 groups as well as to assess the feasibility of the study protocol and acceptability of the Txt2Prevent intervention.

## Methods

### Study Design

The Txt2Prevent study is a mixed method, assessor-blinded, randomized controlled trial with a parallel group design. The study protocol and intervention development have been previously reported [26]. This study is reported in accordance with the Consolidated Standards of Reporting Trials (CONSORT) eHealth checklist [27] and is registered at ClinicalTrials.gov (NCT02336919).

### Participants

Patients with a diagnosis of ACS, as identified by clinical staff, were recruited from St. Paul’s Hospital, a tertiary care hospital, in Vancouver, Canada between June 2015 and October 2016. Patients were eligible to participate if they had ACS (unstable angina or any type of myocardial infarction) as their primary admitting diagnosis, had daily access to a phone with SMS text messaging capabilities, were able to provide informed consent, and were able to read and understand English. Exclusion criteria comprised having coronary artery bypass graft surgery as a treatment for the ACS admission, having a prescheduled surgery within the study period, an expectation that the individual would not survive the duration of the study due to non-CVD reasons, being discharged to a long-term care center, or living outside the province of British Columbia. As this was a pilot study, the sample size was based on convenience. We aimed to recruit 76 participants as we previously estimated this was feasible over 6 months of recruitment. All participants provided written informed consent. Ethics and institutional approvals were obtained from Providence Health Care Research Ethics Board and Simon Fraser University’s Office of Research Ethics.

Baseline questionnaires, which included demographic information as well as measures of self-management, health-related quality of life, and cardiac self-efficacy, were administered in-person in the hospital when possible or within 7 days after discharge. Clinical information was gathered from the participant’s medical record.

### Randomization

After participants completed the baseline questionnaires and were discharged from the hospital, they were randomly assigned to either the intervention (Txt2Prevent plus usual care) or usual care. A statistician not associated with the study generated a random allocation schedule, which randomized participants in a 1:1 ratio using variable block sizes, stratified by sex. A research assistant not involved in recruitment or outcome assessment accessed a secure randomization database to obtain allocations for each patient and informed participants of their group assignment.

### Intervention and Usual Care

Participants in the Txt2Prevent group received 48 unique, automated, one-way messages over 60 days following randomization in addition to usual care. An additional 4 messages relating to study administration (eg, indicating the end of the study and requesting participants to inform us if they were readmitted; see SMS text messages sent on days 7, 26, 45, and 60 in Multimedia Appendix 1) were also sent during the study period. The SMS text messages were delivered at a time of day specified by the participant, began after the participant was randomized, and were sent daily for the first 36 days and then every other day until day 60. We had received feedback from patients in the design stage that it would be helpful to have more support in the beginning, but that daily messages may not be required for the whole period. We primarily covered time-sensitive information about recovery in the first 36 days. Once we covered the primary recovery topics suggested by our clinical advisory committee, we then switched to focus more on healthy living SMS text messages for the remainder of the study period. We chose 60 days for the program length because the initial period after discharge is the highest risk for readmission [4,28]. In addition, many patients will have seen their cardiologist or started a cardiac rehabilitation program by then [29] as well as may be starting to adjust to their new normal or have returned to work [30,31]. SMS text messages covered a range of topics, from time-sensitive information regarding their recovery (eg, timely follow-up with their health care professional) to general healthy living advice (eg, SMS text messages regarding physical activity, diet, and psychosocial health), and were delivered in a prespecified order (Table 1 and Multimedia Appendix 1). Participants received different SMS text messages on 2 occasions based on their smoking status; no other aspects were personalized. The usual care group did not receive any SMS text messages or have any contact from research staff during the study period.

**Table 1:** Examples of the SMS text messages in the intervention group (Txt2Prevent).

Topic	Example SMS text message
Appointment reminders	T2P: See a heart specialist (a cardiologist or internist) within 6 weeks of discharge. If this isn’t set up, call their office, or your family doctor. (Day 15)
Smoking cessation	T2P: Not smoking is one of the most important things you can do for your health. For quitting resources, check out: http://bit.ly/quitnowbc (Day 8)
Recovery guidelines	T2P: Resuming sex: A general guide is that if you can go up a flight of stairs without symptoms, it is probably safe to restart sexual activities. (Day 14)
Psychosocial	T2P: It is common to feel sad or depressed after a heart attack or being in the hospital. If you feel this way for 2+ weeks, contact your doctor. (Day 16)
Physical activity	T2P: Have you done something physically active today? If you have questions, call the Physical Activity Line at 1-877-725-1149 or talk to your doctor. (Day 21)
Medication	T2P: Bring a list of your medications to your appointment when you see your doctor. You can get copies from your pharmacist. (Day 9)

### Outcome Measures and Data Collection

The primary outcome was follow-up scores (controlled for baseline scores) in self-management domains as measured by the Health Education Impact Questionnaire (heiQ; version 3) [32]. The heiQ comprises 40 questions that cover 8 domains in total. All domains were measured and reported on separately: Health-directed behavior (4 questions), Positive and active engagement in life (5 questions), Emotional distress (6 questions), Self-monitoring and insight (6 questions), Constructive attitudes and approaches (5 questions), Skill and technique acquisition (4 questions), Social integration and support (5 questions), and Health service navigation (5 questions). As per the questionnaire’s scoring instructions, each domain score was calculated by averaging Likert scale responses (scaled from 1 to 4). Higher values are desirable, except for the Emotional distress domain. The heiQ was developed using item response theory and structural equation modeling, and the subscales have “acceptable” to “high” internal consistency (Cronbach α ranging from .70 to .86, depending on the domain) [32].

The secondary outcomes were health-related quality of life, cardiac self-efficacy, medication adherence, and health care resource use. Health-related quality of life was measured with the EQ-5D-5L [33], using health state valuations derived from a representative sample of the Canadian adult general population [34]. Self-reported health status was also captured using the EQ-5D-5L Visual Analog Scale (EQ VAS), a 0-100 VAS with anchors defined as “the best health you can imagine” (100) and “the worst health you can imagine” (0) [35]. The EQ-5D has been validated and used around the world. We used the 5-level scale as it has higher discriminatory power, interobserver reliability, and test–retest reliability than the 3-level EQ-5D [33,36]. The 3-level scale was validated in an ACS population, and had high usability, reasonable criterion validity when compared with other quality of life scales, and good test–retest reliability [37]. Cardiac self-efficacy was measured with a modified Sullivan Cardiac Self-Efficacy scale (CSE) [38], such that scores were calculated for the 2 domains (Control symptoms and Maintain function) as well as for the total by averaging Likert scale responses (0-4). For the modifications, we combined the first 4 questions regarding symptom control into 2 questions, as well as added 3 questions about diet and emotional well-being. We calculated the total for the original questions (Total) as well as a total including the additional questions (Total plus). The original Sullivan CSE scale has high internal consistency (Cronbach α of .90 and .87 for the 2 scales, respectively), and good convergent and discriminant validity when compared with other distress and disability scales [38]. For our modifications, Cronbach α were .71, .76, .79, and .82 for “Control symptoms,” “Maintain function,” “Total,” and “Total plus,” respectively. Medication adherence was measured with the Morisky Medication Adherence Scale (MMAS-8) [39-41]. As per questionnaire documentation, we calculated an adherence score on a scale from 1 to 8 and categorized participants as having low (<6), medium (6 to <8), or high adherence (8). This 8-item medication adherence scale has good internal consistency (Cronbach α=.83) and reliability when assessed in a hypertensive population [39]. It has good sensitivity (93%) and moderate specificity (53%) [39]. We also assessed how many participants at follow-up reported taking the recommended medications for post-ACS treatment [42]: acetylsalicylic acid, ticagrelor/clopidogrel, a statin, a beta blocker, and an angiotensin-converting-enzyme inhibitor/angiotensin receptor blocker for those with reduced vascular function. Health care resource use over the 60-day follow-up period (eg, visits to health care practitioners, visits to hospitals, cardiac rehabilitation program participation) was self-reported through a questionnaire developed by the research team. Any self-reported hospitalizations were verified with hospital records. Two blinded assessors (MM and MT) categorized hospital readmissions as cardiac or noncardiac.

Study feasibility was assessed through descriptive statistics on recruitment rates, follow-up rates, questionnaire completion rates, method of questionnaire completion (eg, postal mail, phone), percentage of participants randomized within 7 days, and percentage of participants who completed follow-up within 6 weeks after finishing the 60-day study period. In addition, study staff kept a log of barriers encountered. Acceptability was measured via two 5-level Likert scale survey questions that asked how satisfied participants were with the program (strongly disagree to strongly agree) and whether they thought the program helped them manage their condition. Acceptability was also assessed via 2 questions in semistructured phone interviews—specifically whether they would recommend the program to other patients with ACS and whether they read the SMS text messages. Participants with a range of demographic characteristics who were randomized to the Txt2Prevent group were invited to participate in the semistructured interviews after the 60-day study period. Detailed findings from the interviews, which covered participants’ experiences with the program and gathered feedback on program attributes, will be presented in a separate paper.

Follow-up questionnaires were administered 60 days after randomization primarily via postal mail, except for the health care resource use questions, which were completed over the phone for most participants due to its complex branching. All surveys were administered at baseline and at follow-up, except for the medication adherence scale and health care resource use questionnaire, which were only administered at follow-up.

### Statistics

As this was a pilot study, we undertook similar analyses to what is anticipated for a full trial and considered them exploratory in nature. Descriptive statistics are presented as the mean and SD, or count data with percentages. Analyses were conducted following the intent-to-treat principle. Only complete cases were analyzed. For continuous data from the heiQ, EQ-5D-5L, EQ VAS, and CSE questionnaires, analysis of covariance (ANCOVA) was used to test the effect of group assignment on the follow-up scores when controlling for baseline scores. We then reran the ANCOVA adjusting for age and sex as prespecified covariates as well as previous CVD status for the heiQ, and previous CVD status and marital status for the cardiac self-efficacy, due to their prognostic value [43]. We tested for the following assumptions for ANCOVA tests: independence of covariate and treatment effect, homogeneity of regression slopes, linearity, normality of residuals, homoscedasticity, and homogeneity of variances. The CSE scales, the EQ-5D-5L, and the EQ VAS were negatively skewed, which was primarily driven by outliers. We conducted the analyses with and without the outliers and present results of both analyses in situations where the outlier impacted the conclusion. We also calculated Cohen *d* effect sizes (mean difference score from the Txt2Prevent group minus mean difference score for the usual care group divided by pretest pooled SD [44]) to provide more context for the continuous data results. For our questionnaires, a negative Cohen *d* effect size implies the Txt2Prevent group had worse outcomes than the usual care group.

For count data from the health care resource use questionnaire (eg, number of readmissions), we used negative binomial regression analyses as our data had some overdispersion. For these analyses, we adjusted for age and sex, as prespecified. For binary response data from the health care resource use questionnaire (eg, cardiologist visit within 60 days—yes/no), we used a robust Poisson regression to determine relative risk as our outcomes occurred frequently [45]. For the robust Poisson regression, we adjusted for age and sex as prespecified, as well as geographic region [6] and income [6]. Chi-square tests were used for categorical variables unless there were low expected counts, in which case the Fisher exact test was used. Analyses were performed using SPSS (version 25; IMB Corp). Statistical significance was set at *P*<.05.

## Results

### Participant Demographics

**Figure 1:**
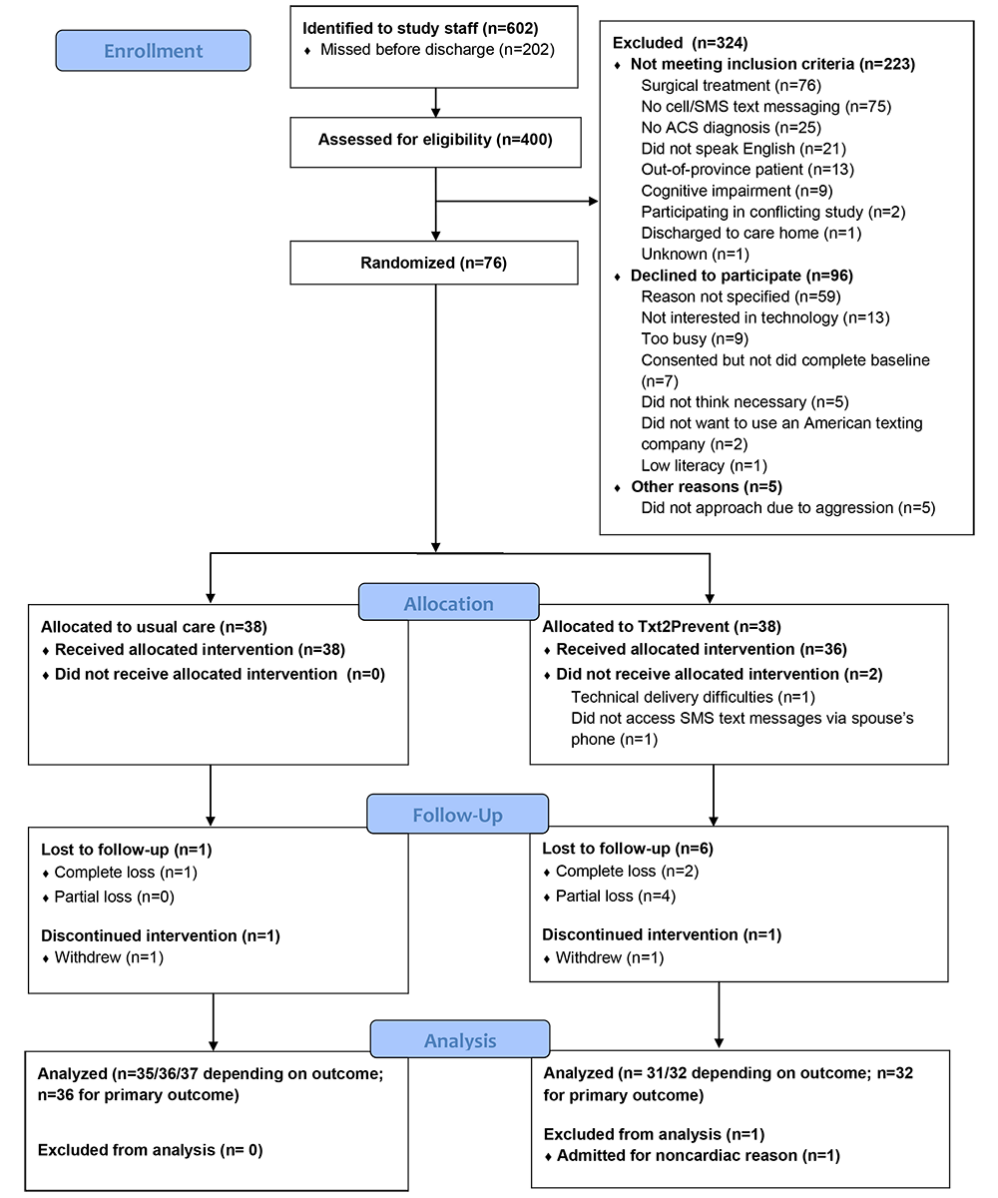
CONSORT flow diagram.

Four hundred patients were assessed for eligibility from June 2015 to October 2016. After excluding those who did not meet inclusion criteria and those who declined to participate, 76 participants were randomized (Figure 1).

The mean age of participants was 60 years (SD 9.3) and 73% (55/75) were male (Tables 2 and 3). Nine participants did not complete the study (2 withdrew, 3 failed to complete any of the follow-up questionnaires, and 4 partially completed the follow-up questionnaires; Figure 1). There were no statistically significant differences in baseline characteristics between those who did not complete the follow-up assessments compared with those who did, including in age (t_73_=–0.16 [2 tailed], *P*=.87), sex (Fisher exact test, *P*=.18), and whether or not they completed education past high-school (Fisher exact test, *P=*.09; full analyses not shown). Data collection ended in December 2016.

**Table 2:** Baseline demographics, by group.

Variable					Group^a^			
					Txt2Prevent			Usual care
**Socioeconomic status**								
	Age, mean (SD)			59.5 (9.1)			61.1 (9.6)	
	Male, n (%)			27 (73)			28 (74)	
	Married (yes/no), n (%)			27 (73)			31 (82)	
	**Geographic region, n (%)**							
		Census metropolitan area (100,000+ urban core) [46]	13 (35)			21 (55)		
		Census agglomeration (10,000-99,999 urban core) [46]	18 (49)			8 (21)		
		Rural [46]	6 (16)			9 (24)		
	Greater than high-school education (yes/no), n (%)			23 (62)			25 (66)	
	Employed full-time (yes/no), n (%)			20 (54)			17 (45)	
	**Household income, n/N (%)^b^**							
		Less than Can $29,999^a^	6/33 (18)			7/36 (19)		
		Can $30,000 to Can $69,999	9/33 (27)			7/36 (19)		
		Can $70,000 to Can $99,999	6/33 (18)			6/36 (17)		
		Can $100,000 or higher	12/33 (36)			16/36 (44)		
**Technology use, n/N (%)^b^**								
	At least daily cell use			26/36 (72)			34/38 (89)	
	Very or completely confident using a cell phone			18/33 (55)			26/37 (70)	
	Own a smartphone			34/37 (92)			33/37 (89)	
**Comorbidities and medical history, n (%)**								
	Hypertension			25 (68)			19 (50)	
	Dyslipidemia			18 (49)			12 (32)	
	Diabetes (type 1 or type 2)			14 (38)			7 (18)	
	Previous any type of cardiovascular disease			16 (43)			16 (42)	
**Treatment in hospital, mean (SD)**								
	Days in hospital			5.1 (3.0)			5.2 (4.2)	
	**Primary reason for admission, n (%)**							
		Non-ST-segment elevation acute coronary syndrome	10 (27)			18 (47)		
		ST-segment elevation myocardial infarction	22 (59)			17 (45)		
		Other	5 (14)			3 (8)		
	Revascularization, n (%)			29 (78)			33/37 (89)	
	Current/quit within 6-month smoker, n (%)			8 (22)			8/37 (22)	
**Medication at discharge, n/N (%)**								
	Acetylsalicylic acid			33/36 (92)			36/37 (97)	
	Ticagrelor or clopidogrel			31/36 (86)			36/37 (97)	
	Statin			33/36 (92)			33/37 (89)	
	Beta blocker			29/36 (81)			33/37 (89)	
	Angiotensin-converting-enzyme inhibitor or angiotensin II receptor blocker			29/36 (81)			32/37 (86)	

^a^n=37 and =38 for the Txt2Prevent and Usual care groups unless stated otherwise.

^b^Can $1 = US $0.81.

**Table 3:** Baseline questionnaire scores, by group.

Baseline variables, mean (SD)				Group	
				Txt2Prevent (n=37)	Usual care (n=38)
**Health Education Impact Questionnaire (heiQ)**					
	Health-directed activity		2.93 (0.80)		2.93 (0.78)
	Positive and active engagement in life		3.13 (0.53)		3.27 (0.48)
	Emotional distress		2.25 (0.68)		2.02 (0.60)
	Self-monitoring and insight		3.19 (0.89)		3.08 (0.59)
	Constructive attitudes and approaches		3.17 (0.51)		3.35 (0.46)
	Skill technique and acquisition		3.02 (0.34)		3.09 (0.53)
	Social integration and support		3.11 (0.49)		3.30 (0.41)
	Health service navigation		3.08 (0.47)		3.27 (0.47)
EQ-5D-5L				0.833 (0.119)	0.849 (0.109)
EQ-5D-5L Visual Analog Scale (EQ VAS)				67.00 (19.00)	68.00 (17.00)
**Cardiac Self-Efficacy (CSE)**					
		Symptoms	3.14 (0.63)		3.25 (0.52)
		Function	2.85 (0.91)		2.91 (0.83)
		Total	3.02 (0.61)		3.10 (0.57)
		Total plus	2.95 (0.58)		3.02 (0.54)

### Primary Outcome

There were no statistically significant differences between groups for the heiQ scores in either the unadjusted (Multimedia Appendix 2) or adjusted model in any of the 8 domains (adjusted mean difference [Txt2Prevent minus usual care] for each domain: Health-directed activity: –0.13, 95% CI –0.39 to 0.13, *P*=.31; Positive and active engagement in life: 0.03, 95% CI –0.19 to 0.25, *P*=.76; Emotional distress: 0.04, 95% CI –0.22 to 0.29, *P*=.77; Self-monitoring and insight: –0.14, 95% CI –0.33 to 0.05, *P*=.15; Constructive attitudes and approaches: –0.10, 95% CI –0.36 to 0.17, *P*=.47; Skill technique and acquisition: 0.05, 95% CI –0.18 to 0.27, *P*=.69; Social integration and support: –0.12, 95% CI –0.34 to 0.10, *P*=.27; Health services navigation: –0.05, 95% CI –0.29 to 0.19, *P*=.69) (Table 4). Cohen *d* effect sizes were all below 0.20, indicating negligible effects, except for the Self-monitoring and insight domain, where the Txt2Prevent group had worse outcomes than the usual care group, estimated at a small negative effect (*d*=–0.48; Table 4).

**Table 4:** Adjusted 60-day Health Education Impact Questionnaire (heiQ) scores, by group.^a^

Outcome		Group		Adjusted mean difference (95% CI)	*P* value	Effect size (Cohen *d*)^b^
		Txt2Prevent (n=32), adjusted mean (95% CI)	Usual care (n=36), adjusted mean (95% CI)			
**Health Education Impact Questionnaire (heiQ)**						
	Health-directed activity^c^	3.02 (2.82 to 3.21)	3.15 (2.96 to 3.35)	–0.13 (–0.39 to 0.13)	.31	–0.15
	Positive and active engagement in life	3.10 (2.93 to 3.26)	3.06 (2.91 to 3.22)	0.03 (–0.19 to 0.25)	.76	0.10
	Emotional distress	2.37 (2.18 to 2.56)	2.33 (2.15 to 2.51)	0.04 (–0.22 to 0.29)	.77	–0.05
	Self-monitoring and insight	3.08 (2.94 to 3.23)	3.22 (3.09 to 3.36)	–0.14 (–0.33 to 0.05)	.15	–0.48
	Constructive attitudes and approaches	3.09 (2.89 to 3.29)	3.18 (2.99 to 3.38)	–0.10 (–0.36 to 0.17)	.47	–0.06
	Skill technique and acquisition	2.91 (2.73 to 3.08)	2.86 (2.70 to 3.03)	0.05 (–0.18 to 0.27)	.69	0.14
	Social integration and support	3.04 (2.87 to 3.21)	3.17 (3.01 to 3.33)	–0.12 (–0.34 to 0.10)	.27	–0.04
	Health services navigation	3.15 (2.97 to 3.33)	3.19 (3.02 to 3.37)	–0.05 (–0.29 to 0.19)	.69	0.15

^a^The adjusted heiQ model includes baseline scores, age, sex, and previous cardiovascular disease status (yes/no).

^b^Effect size is Cohen *d* (mean difference score from the Txt2Prevent group minus mean difference score for the usual care group divided by pretest pooled SD [44]). For our questionnaires, a negative number implies the Txt2Prevent group had worse outcomes than the usual care group.

^c^In the usual care group, 35 participants were analyzed for the Health-directed activity domain.

### Secondary Outcomes

There were no statistically significant differences in EQ-5D-5L health state values (*P*=.51) or EQ VAS scores (*P*=.71; Table 5). For cardiac self-efficacy, in the adjusted models, there were no statistically significant differences between the 2 groups, except for the “Total plus” domain, where the Txt2Prevent group had worse outcomes (Control symptoms: *P*=.10; Maintain function: *P=*.05; Total: *P*=.05; Total plus: *P*=.03; Table 5). The statistically significant finding on the “Total plus” scale was due to an influential outlier that impacted the normality assumptions of ANCOVA. When the influential outlier was excluded in the adjusted analysis, the *P* value for the Total plus scale was no longer significant (*P*=.05). Depending on the self-efficacy domain, there were small or medium negative effects for the Txt2Prevent group (ie, this group had worse outcomes) based on the Cohen *d* values for the self-efficacy scores (Table 5).

**Table 5:** Adjusted 60-day EQ-5D-5L Visual Analog Scale (EQ VAS), EQ-5D-5L, and Cardiac Self-Efficacy results, by group.^a^

Outcome			Group				Adjusted mean difference (95% CI)				Adjusted *P* value			Effect size (Cohen *d*)^b^
			Txt2Prevent (n=32), adjusted mean (95% CI)		Usual care (n=36), adjusted mean (95% CI)									
EQ-5D-5L Visual Analog Scale (EQ VAS)			70.94 (65.91 to 75.98)		69.68 (64.84 to 74.52)		–1.27 (–5.41 to 7.94)			.71			0.10	
EQ-5D-5L^c^			0.82 (0.78 to 0.86)		0.84 (0.80 to 0.88)		–0.018 (–0.07 to 0.04)			.51			–0.13	
**Cardiac Self-Efficacy Scale (CSE)**														
	Control symptoms	2.49 (2.24 to 2.75)		2.76 (2.49 to 3.02)		–0.27 (–0.58 to 0.05)				.10			–0.43	
	Control symptoms (2 outliers removed)^c^	2.57 (2.36 to 2.78)		2.80 (2.57 to 3.02)		–0.23 (–0.49 to 0.04)				.09			–0.37	
	Maintain function	2.14 (1.84 to 2.45)		2.52 (2.20 to 2.84)		–0.38 (–0.76 to 0.004)				.05			–0.46	
	Maintain function (1 outlier removed)^c^	2.23 (1.95 to 2.50)		2.50 (2.22 to 2.78)		–0.27 (–0.61 to 0.07)				.11			–0.35	
	Total	2.35 (2.09 to 2.60)		2.66 (2.39 to 2.93)		–0.31 (–0.63 to 0.003)				.05			–0.55	
	Total (1 outlier removed)^c^	2.42 (2.20 to 2.64)		2.64 (2.41 to 2.86)		–0.22 (–0.49 to 0.05)				.11			–0.40	
	Total plus	2.28 (2.03 to 2.53)		2.64 (2.38 to 2.90)		–0.36 (–0.66 to –0.05)				.03			–0.65	
	Total plus (1 outlier removed)^c^	2.35 (2.14 to 2.57)		2.62 (2.39 to 2.84)		–0.26 (–0.53 to 0.003)				.05			–0.51	

^a^The adjusted EQ-5D-5L and EQ VAS models include baseline scores, age, and sex. The adjusted CSE model includes baseline scores, age, sex, marital status, and previous cardiovascular disease status (yes/no).

^b^Effect size is Cohen *d* (mean difference score from the Txt2Prevent group minus mean difference score for the usual care group divided by pretest pooled SD [44]). For our questionnaires, a negative number implies the Txt2Prevent group had worse outcomes than the usual care group.

^c^In the Txt2Prevent group, 31 participants were analyzed for the EQ-5D-5L questionnaire. Thirty-two participants were analyzed for the remaining outcomes (excluding those with outliers removed). For the 2 outliers in the Control symptoms domain, 1 was from the Txt2Prevent group and 1 from the usual care group. The 1 outlier for the Maintain function, Total, and Total plus was from the Txt2Prevent group.

There were no statistically significant differences in the mean medication adherence scores between the 2 groups (*P*=.27; Multimedia Appendix 2). When categorized into low, medium, and high adherence, 34% (11/32) of those in the Txt2Prevent group and 42% (15/36) of those the usual care group were classified as high adherers (χ^2^_2_=2.10, *P*=.35). There were no statistically significant differences between the 2 groups for the categories of cardiac medications they were prescribed (Fisher exact test; *P* values ranged from .24 for statins to >.99 for beta blockers; Multimedia Appendix 3).

There were no differences between the groups in either the percentage of participants who visited the hospital or the mean number of visits to the hospital for all-cause or cardiac visits (Table 6). There were no differences in whether participants had visited a family physician, joined a cardiac rehabilitation program, or visited a cardiologist over the study period in adjusted analyses (Multimedia Appendix 4).

**Table 6:** Type of and mean hospital visits within 60 days, by group.

Outcome	Group^a^			*P* value	Group^b^				*P* value
	Txt2Prevent (n=32), adjusted mean visits (95% CI)	Usual care (n=37), adjusted mean visits (95% CI)			Txt2Prevent (n=32), n (%)	Usual care (n=37), n (%)			
Cardiac emergency department	0.00 (–)	0.08 (0.02-0.38)	N/A		0 (0)	3 (8)		.24	
All-cause emergency department	0.20 (0.08-0.48)	0.33 (0.16-0.66)	.36		3 (9)	9 (24)		.10	
Cardiac hospitalization	0.13 (0.04-0.37)	0.12 (0.04-0.34)	.92		3 (9)	3 (8)		1.00	
All-cause hospitalization	0.16 (0.06-0.42)	0.21 (0.09-0.48)	.70		4 (13)	6 (16)		.74	

^a^Mean visits were analyzed with a negative binomial regression adjusted for age and sex.

^b^Number of participants—n (%)—admitted for all-cause emergency department visits was analyzed with chi-square (*df*=1) while the remaining visit types were analyzed with a Fisher exact test due to low expected cell counts.

### Assessment of Feasibility and Acceptability

Recruitment of the target sample took 17 months (Table 7), which was longer than the anticipated 6 months. As much as 55.8% (223/400) of patients we approached were ineligible (Figure 1), of which 34.1% (76/223) were scheduled for surgery and 33.6% (75/223) did not own a cell phone. Of those eligible, 55.8% (96/172) declined to participate (Figure 1). Our randomization system worked well as 97% (74/76) of participants were randomized within our target of 7 days of discharge and 66% (50/76) were randomized within 2 days (Table 7). Those that were not randomized within the target time frame were due to delayed completion of baseline questionnaires. Almost 91% of participants (69/76) completed follow-up for the primary outcome. We obtained all follow-up questionnaires for 88% (67/76) of participants and at least one follow-up questionnaire for 93% (71/76) of participants; however, one participant was excluded from the analysis because they were admitted for a noncardiac reason. While we developed electronic versions to provide an alternative option, the majority of participants were willing to complete the questionnaires in their default format (60/69 [87%] completed packaged questionnaires by postal mail and 65/69 [94%] completed the health care resource use by telephone; Table 7).

We had a technical problem with our delivery system 11 months into recruitment where 80 SMS text messages were not delivered for 10 days for 28% (10/36) participants. It is suspected an operating system update caused the error as a server reboot fixed the error. Once we fixed the error, all affected participants resumed the SMS text messages where they left off. In order to keep blinding and consistency in the timing of the outcome measurements, follow-up assessments were still scheduled for 60-days after randomization. Of the 10 affected participants, 2 did not complete follow-up, 5 completed the primary outcome assessment between 60 and 70 days after randomization, and 3 completed follow-up after 70 days. After this technical problem, we implemented more regular system checks by the staff involved in randomization to ensure all SMS text messages were being delivered.

Regarding acceptability, over 93% (29/31 [94%]) of participants in the Txt2Prevent group reported they agreed or strongly agreed that they were satisfied with the program. About 74% (23/31) agreed or strongly agreed that it helped them manage their condition. When asked in semistructured interviews, 17/18 participants said they would recommend the program to other patients with cardiac issues. The participant who said he would not recommend the program stated that his recommendation would depend on whether the person took the time to read the SMS text messages. All but 2 interview participants reported reading every SMS text message, and these reported that while they did read most of the SMS text messages, it was possible they did not read all of them. All interviewed participants said they would be willing to use SMS text messaging again for health purposes.

**Table 7:** Study protocol feasibility measures.^a^

Feasibility measure		Descriptive assessment
**Recruitment**		
	Months of recruitment	17
	Number participants randomized per month, mean (range)	4.5 (0-15)
	Ineligible patients, n/N (%)	223/400 (55.8)
	Eligible patients who declined to participate, n/N (%)	96/172 (55.8)
**Randomization**		
	Days from discharge to randomization, mean (SD)	2.1 (2.1)
	Participants randomized within 7 days of discharge, n/N (%)	74/76 (97)
**Follow-up**		
	Completed packaged follow-up questionnaires, n/N (%)	69/76 (91)
	Packaged follow-up questionnaires done by postal mail, n/N (%)	60/69 (87)
	Days after discharge to complete packaged follow-up questionnaires, mean (SD)	73 (17)
	Completed packaged follow-up questionnaires done within 6 weeks of the 60-day study period, n/N (%)	64/69 (93)
	Completed health care resource use follow-up questionnaires, n/N (%)	69/76 (91)
	Health care resource use questionnaires by phone, n/N (%)	65/69 (94)
	Days after discharge to complete health care resource use questionnaire, mean (SD)	69 (14)
	Completed health care resource use questionnaires done within 6 weeks of the 60-day study period, n/N (%)	67/69 (97)
	Participants who completed all sets of follow-up questionnaires, n/N (%)	67/76 (88)
	Participants who completed no follow-up questionnaires, n/N (%)	5/76 (7)
	Questions completed on received questionnaires, n/N (%)	4613/4624 (99.8)

^a^Packaged follow-up questionnaires included Health Education Impact Questionnaire, Cardiac Self-Efficacy Scale, Morisky Medication Adherence Scale, EQ-5D-5L, and EQ-5D-5L Visual Analog Scale.

## Discussion

### Principal Findings

Our pilot study assessed the impact, feasibility, and acceptability of a 60-day SMS text messaging program in supporting patients with ACS following hospital discharge. In exploratory adjusted analyses, we did not find statistically significant differences on follow-up scores (controlling for baseline scores where applicable) between the Txt2Prevent group and the usual care group in their self-management domains, health-related quality of life, medication adherence, health care resource use, and self-efficacy in adjusted models, except for the “Total plus” domain, which was impacted by an influential outlier. The study protocol was generally feasible, as seen by high adherence to the study protocol targets for randomization time frames and questionnaire completion rates, although recruitment took much longer than estimated. In terms of acceptability, participants reported they generally found the program acceptable and believed it helped them manage their condition.

In our pilot study, we failed to demonstrate the effect of SMS text messaging on our questionnaire outcomes, including the heiQ, CSE, and medication adherence. Previously, 2 interventions using apps reported improvements in heiQ domains over the short term [47,48], although this was not the case in 2 web-based interventions [49,50]. We also did not find improvements in self-efficacy scores, which is in contrast to a study that used SMS text messages and phone calls for patients with CVD [51]. In addition, several previous SMS text messaging studies targeting CVD medication adherence had a positive effect [52]. Other studies assessing SMS text messaging in a CVD population have measured specific risk factors, such as blood pressure and cholesterol. Chow et al [24] reported positive effects on low-density lipoprotein–cholesterol, systolic blood pressure, body mass index, physical activity, and smoking in an SMS text messaging program. However, not all studies have reported positive effects [22]. Zheng et al [53], who used a framework similar to Chow et al’s [24], found greater levels of physical activity at 6 months in the intervention group compared with the control group, but did not observe statistically significant (*P*=.22) effects on blood pressure. Another study investigating the effect of weekly SMS text messages or emails on the primary prevention of CVD risk factors found no improvements after 1 year [54]. Therefore, while many previous studies have reported positive effects, the results are not consistent.

Differences between our findings and others may be due to our intervention’s design. Guided by an advisory committee that included clinicians (a cardiac nurse, a family physician, a community pharmacist, and 2 cardiologists), researchers, and 2 patient partners with lived experiences of CVD, the SMS text messages were education based and included prompts that aligned with hospital messaging and current guidelines [55]. The messages were revised according to feedback from patient focus groups. We wanted to test whether a simple program design (ie, one-way delivery, prespecified order of SMS text messages) was effective before considering more complicated interventions. SMS text messages were designed to broadly apply to patients and did not include tailoring, such as personalization, feedback, or content matching. Incorporating tailored messages could be beneficial as systematic reviews identified that mobile health studies with positive effects often used tailoring [56,57]. In addition, by having a multifactorial focus, we may not have covered topics frequently enough to instigate change. For example, our medication adherence results contrast previous studies reporting positive effects [22], but only 7 of our 48 SMS text messages covered medication*-*related topics (see SMS text messages sent on day 1, 7, 9, 17, 18, 22, and 24 in Multimedia Appendix 1). The messages were also 1 way only, in part because this required fewer resources to implement. This may be a limitation as some meta-analyses and reviews have reported 2-way messages were more effective, although this is not consistent [57-59]. Chow et al [60] incorporated behavior change techniques such as prompting consequences and self-monitoring of behavior. It is unclear which behavior change techniques are effective, but a future study could consider including more behavior change techniques [61,62].

Regarding the feasibility of the study protocol, we required 17 months to recruit 76 participants instead of the anticipated 6 months. Six months was estimated because there were approximately 750 ACS discharges in the previous year, and a feasibility survey showed 50% (14/28) of patients owned a mobile phone. We assumed 40.0% (300/750) of patients would be eligible and of those 50.0% (150/300) would agree to participate. However, we missed approaching many patients due to restrictions imposed by our ethics board. The research assistant had to obtain bed numbers of patients with ACS from the clinical nurse leader. They then asked the bedside nurse to confirm with the patient if they could explain the study. This required forming strong relationships with clinical staff. Evening recruitment visits also helped as patients were often discharged shortly after returning to the ward from the catheterization laboratory. Ultimately, 43.0% (172/400) of approached patients were eligible and 44.2% (76/172) of eligible patients agreed to participate. More patients declined to participate in our study compared with the 10%-30% refusal rates reported by other CVD SMS text messaging studies that recruited from hospitals or outpatient clinics [23,63]. Many patients refused when initially approached by clinical staff, so we could not document their reasons. Having brief, standardized wording to provide clinical staff could increase uptake. Although the focus groups and feasibility survey indicated patients were interested in this type of program, the hospital environment could have created challenges as patients may be overwhelmed, unsure of their postdischarge needs, or be wary of committing to a research project [64]. However, recruiting outside the hospital would contradict with the time-sensitive nature of the program. The randomization process worked well, although delays happened when questionnaires could not be completed before discharge. Our follow-up rates were slightly lower than previous CVD SMS text messaging studies, which were often between 97% and 91% [53,63,65,66]. Having 2 different questionnaire formats (telephone for 1 questionnaire and mail for the remaining questionnaires) likely caused some of the partial completions.

### Limitations

Our study has several limitations to consider. As this was a pilot study, we did not determine our sample size based on power calculations and were likely underpowered to detect clinically important differences. We chose the heiQ as it covers potential proximal and intermediate outcomes of self-management programs [32]. Self-management is important as it is linked to improved health behaviors and reduced costs and health care visits [67-69]. Previously, a 10-week proof-of-concept study (n=35) evaluating a peer-support app reported improvements in heiQ domains, indicating changes are possible in small samples over the short term [48]. Other SMS text messaging studies frequently use clinical measures, such as blood pressure, making it difficult to compare our results directly. In addition, as our measures were self-reported, there may have been biases (eg, social desirability bias or recall bias); however, we primarily used validated questionnaires and confirmed self-reported hospital visits with hospital records. For some measures, a clinically meaningful change has not yet been determined, so we calculated Cohen *d* effect sizes for better comparison [70]. Participants in the intervention group may have been impacted in ways not captured by the questionnaires or had a different perspective at follow-up (eg, been more aware they were not meeting recommendations). While we cannot know this, it is possible as participants in the interviews and acceptability survey provided positive feedback about the program.

### Conclusions

In our exploratory analyses, we did not demonstrate any positive effects of the SMS text messaging intervention in terms of self-management, medication adherence, health-related quality of life, cardiac self-efficacy, or health care resource use. The Txt2Prevent program had an intentionally simple design and was acceptable to participants, but design changes may be needed before proceeding to a larger study. The study protocol was feasible to implement, although improvements to the recruitment process are likely required. Future work should investigate the effect of tailoring, multifactor versus single-factor interventions, 2-way versus 1-way SMS text messaging, and the effectiveness of behavior change techniques.

## Appendix

Multimedia Appendix 1 Txt2Prevent intervention text messages.

Multimedia Appendix 2 Additional analyses - unadjusted Health Education Impact Questionnaire (heiQ), EQ-5D-5L, EQ-5D-5L Visual Analog Scale (EQ VAS), Cardiac Self-Efficacy (CSE), and medication adherence results at 60-days (controlling for baseline scores).

Multimedia Appendix 3 Additional analyses - 60-day follow-up medication prescriptions, by group.

Multimedia Appendix 4 Additional analyses - physician visits and cardiac rehabilitation enrolment within 60-days, by group.

Multimedia Appendix 5 CONSORT E-HEALTH checklist (v1.6.1).

## Abbreviations

ACS: acute coronary syndrome
ANCOVA: analysis of covariance
CSE: Cardiac Self-Efficacy scale
CVD: cardiovascular disease
EQ VAS: EQ-5D-5L Visual Analog Scale
MMAS: Morisky Medication Adherence Scale
