# Supplementary material for: The Use of SMS Text Messaging to Improve the Hospital-to-Community Transition in Patients With Acute Coronary Syndrome (Txt2Prevent): Results From a Pilot Randomized Controlled Trial
Source: JMIR Mhealth Uhealth. 2021 May 14;9(5):e24530. doi: 10.2196/24530 (PMC8164115; doi:10.2196/24530)
Supplement: Multimedia Appendix 1 [file mhealth_v9i5e24530_app1.docx]

# Appendix 1. Txt2Prevent Study Text Messages

Table 1. Text messages used in the Txt2Prevent program.

| Day | Topic | Message |
| --- | --- | --- |
| 1 | Prescription Reminder | Fill your hospital medication prescriptions as soon as possible. Make sure you know how and when to take all your medications. |
| 2 | Appointment with Family Physician Reminder | Make an appointment to see your family doctor within 2 weeks of leaving the hospital. If you need a doctor, try the tool at: http://bit.ly/findaMD |
| 3 | Physical Activity – Symptoms | If you have chest pain, stop & rest. Take nitro spray if your doctor prescribed it. Call 911 if symptoms are still there in 5 minutes. |
| 4 | Physical Activity | In the early stages of recovery, take rests and try to avoid things like lifting heavy objects. |
| 5 | Help | You can call or access Healthlink BC at 8-1-1 or <http://bit.ly/findaMD> to ask questions you have or to help find resources in your area. |
| 6 | Physical Activity – Cardiac Rehab | We strongly recommend that you join a cardiac rehabilitation program. Contact Healthlink BC or your doctor to find out if a program is near you. |
| 7 | Medication | If you had a stent, it is especially important to take your anti-platelet medications like clopidogrel (Plavix) or ticagrelor (Brilinta). |
| 7 | Readmission Reminder | If you are readmitted to the hospital at any point during the study, let us know at 604-682-2344 ext. 64874. |
| 8 | Smoking Cessation  (Smoking Stream) | Not smoking is one of the most important things you can do for your health. For quitting resources, check out: http://bit.ly/quitnow.bc |
| 8 | Not Smoking (Non-smoking Stream) | Staying smoke-free is one of the most important things you can do for your health. |
| 9 | Medication List to Appointment Reminder | Bring a list of your medications to your appointment when you see your doctor. You can get copies from your pharmacist. |
| 10 | Resting/Taking Breaks | You may feel like you have less energy. It’s okay to take breaks, ask for help, and to say no if you think some activities are too much for right now. |
| 11 | Driving | Check with your doctor about when it is safe for you to start driving again. |
| 12 | Physical Activity | Walk around and be active if you can, but make sure you go at your own pace and are within your abilities. |
| 13 | Communicating with Friends and Family | While you’re recovering, you may want someone else to update friends & family about how you’re doing, such as through phone calls, emails or Facebook. |
| 14 | Sex | Resuming sex: A general guide is that if you can go up a flight of stairs without symptoms, it is probably safe to restart sexual activities. |
| 15 | Specialist Follow Up | See a heart specialist (a cardiologist or internist) within 6 weeks of discharge. If this isn't set up, call their office, or your family doctor. |
| 16 | Depression 1 | It is common to feel sad or depressed after a heart attack or being in the hospital. If you feel this way for 2+ weeks, contact your doctor. |
| 17 | New symptoms/side effects | You may have new and different symptoms or have side effects from your medications. If you have concerns, contact your doctor. |
| 18 | Pharmacist | If you want to go over your medications, contact your pharmacist. You can also see if your pharmacy has automatic refill reminders. |
| 19 | Work | Check with your doctor about when you may go back to work. This can depend on many factors. You may want to start part-time. |
| 20 | Help | If you have questions, write them down as they happen and bring the list to your doctor. You may want to keep a list in a small book. |
| 21 | Physical Activity | Have you done something physically active today? If you have questions, call the Physical Activity Line at 1-877-725-1149 or talk to your doctor. |
| 22 | Medication | Having a hard time remembering to take your medication? Set an alarm, use a pillbox, create a routine or ask the pharmacist for ideas. |
| 23 | Smoking – Quit Date (Smoking Stream) | Setting a quit date is an important step to stop smoking. Have you thought about setting one? If you have recently quit, enjoy your smoke-free life. |
| 23 | Second Hand Smoke (Non-smoking Stream) | Make your home, car and work place smoke-free. Second-hand smoke is harmful, so try to limit your exposure. |
| 24 | Prescription Reminder | If you have any 30-day medication prescriptions, remember to refill them |
| 25 | Air Travel | Travelling? Check with your doctor, insurance & airline. Take a list of your meds, double the amount you’ll need, and put them in your carry-on. |
| 26 | Readmission Reminder | If you are readmitted to the hospital at any point during the study, let us know at 604-682-2344 ext. 64874. |
| 27 | Diet | Diet matters. Try to eat a wide variety of vegetables and fruits every day by adding them to salads, soups, stews, stir-fries and smoothies. |
| 28 | Social Support | Social support groups can be helpful. Consider joining or starting a walking group, a group on Facebook, or a group at your community centre. |
| 29 | Diet | Try to eat foods with lots of fibre in them like whole fruit and vegetables, whole grains, and oatmeal. |
| 30 | Depression 2 | Have you been feeling sad, down or uninterested in life? These, among others like low energy, are symptoms of depression. Ask your doctor. |
| 31 | Diet | Talk to a dietitian about healthy eating by calling 8-1-1 from 9-5 Monday to Friday. You can also email them through the Healthlink BC website. |
| 32 | Anger | Being angry is a normal feeling after a hospitalization. Use stress management techniques like meditation, deep breathing or yoga. |
| 33 | Depression Resources | Try the Antidepressant Skills Workbook to better understand and manage depression: http://www.comh.ca/antidepressant-skills/adult/ |
| 34 | Physical Activity | Have you been doing something physically active every day, like going for a walk? |
| 35 | Sex | Medications for heart conditions can affect sexual desire and bodily functions. If this is an issue, talk to your doctor. |
| 36 | Activities | What things do you enjoy doing? Set aside time to do activities you like or try something new. |
| 37 |  |  |
| 38 | Sleep | Sleep is important. Try to have a regular wake-up time & make sure your bedroom is a comfortable temperature with minimal noise & light. |
| 39 |  |  |
| 40 | Stress | Frequent stress can be harmful. Try to identify the cause, and find activities that help you like yoga, meditation, or deep breathing. |
| 41 |  |  |
| 42 | Stress/coping | Try the “Positive Coping with Health Conditions” handbook to help with managing self-care and stress: http://bit.ly/positivecoping |
| 43 |  |  |
| 44 | Diet | Try to avoid processed meats like deli meats and sausages. Try eating lean white meat, fish, lentils, beans and nuts instead. |
| 45 | Readmission Reminder | If you are readmitted to the hospital at any point during the study, let us know at 604-682-2344 ext. 64874. |
| 46 | Depression 3 | Heart patients who are depressed do not recover as well as patients who aren’t depressed. Get support (family, friends, doctor) if you need it. |
| 47 |  |  |
| 48 | Diet | Try to lower the amount of salt you eat by avoiding processed foods and removing the salt shaker from the table. For flavour, try adding pepper or other spices instead. |
| 49 |  |  |
| 50 | Anxiety | In the past 2 weeks, how much have you been bothered by feeling anxious or nervous (out of 10)? If 5+ , consider seeing your doctor. |
| 51 |  |  |
| 52 | Physical Activity | Are you still being physically active? Add it to daily activities like walking an extra bus stop, taking the stairs, or taking a walking break. |
| 53 |  |  |
| 54 | Diet | How many fruits and vegetables did you eat today? Try cutting them up to have as snacks during the day. |
| 55 |  |  |
| 56 | Social Support | Try to spend time with your family and friends. Having a good social support group can be helpful and important. |
| 57 | Take Control of Health | It's important to take control of your own health. If you have questions, make sure you ask your doctor. |
| 58 | Wrap Up | Try your best to eat healthily, be physically active, take your medications, stop smoking and maintain your mental health. They all help! |
| 59 | Wrap Up | The Txt2Prevent messages will end tomorrow. If you have further questions about your condition, contact your doctor. |
| 60 | Wrap Up | This is the last text of the Txt2Prevent program. Thank you for your participation in the study! You will soon be contacted for follow-up. |
