# Supplementary material for: The Use of SMS Text Messaging to Improve the Hospital-to-Community Transition in Patients With Acute Coronary Syndrome (Txt2Prevent): Results From a Pilot Randomized Controlled Trial
Source: JMIR Mhealth Uhealth. 2021 May 14;9(5):e24530. doi: 10.2196/24530 (PMC8164115; doi:10.2196/24530)
Supplement: Multimedia Appendix 2 [file mhealth_v9i5e24530_app2.docx]

Multimedia Appendix 2. Unadjusted Health Education Impact Questionnaire (heiQ), EQ-5D-5L, EQ-5D-5L Visual Analog Scale (EQ VAS), Cardiac Self-Efficacy (CSE), and medication adherence results at 60-days (controlling for baseline scores).^a^

|  | | Txt2Prevent (n=31/32)^b^, unadjusted mean  (95% CI) - | Usual care (n=35/36)^b^, unadjusted mean  (95% CI), | Unadjusted mean difference  (95% CI) | *P*-value |
| --- | --- | --- | --- | --- | --- |
| Health Education Impact Questionnaire (heiQ) | | | | | |
|  | Health directed activity | 3.08 (2.88 to 3.27) | 3.22 (3.04 to 3.41) | –0.14 (–0.41 to 0.13) | .29 |
|  | Positive and active engagement in life | 3.15 (2.99 to 3.31) | 3.12 (2.97 to 3.27) | 0.03 (–0.20 to 0.25) | .82 |
|  | Emotional distress | 2.29 (2.10 to 2.48) | 2.24 (2.06 to 2.42) | 0.05 (–0.22 to 0.31) | .73 |
|  | Self-monitoring and insight | 3.13 (2.98 to 3.27) | 3.26 (3.12 to 3.39) | –0.13(–0.33 to 0.07) | .21 |
|  | Constructive attitudes and approaches | 3.14 (2.95 to 3.34) | 3.25 (3.07 to 3.43) | –0.10 (–0.37 to 0.16) | .44 |
|  | Skill technique and acquisition | 2.93 (2.76 to 3.10) | 2.90 (2.74 to 3.06) | 0.03 (–0.20 to 0.26) | .80 |
|  | Social integration and support | 3.09 (2.93 to 3.25) | 3.21 (3.06 to 3.36) | –0.12 (–0.35 to 0.10) | .29 |
|  | Health services navigation | 3.20 (3.02 to 3.37) | 3.24 (3.07 to 3.40) | –0.04 (–0.29 to 0.20) | .73 |
| EQ VAS | | 73.10 (67.97 to 78.22) | 72.33 (67.50 to 77.17) | 0.76 (–6.29 to 7.82) | .83 |
| EQ-5D-5L | | 0.84 (0.80 to 0.88) | 0.86 (0.82 to 0.90) | –0.02 (–0.08 to 0.04) | .52 |
| Cardiac Self-Efficacy (CSE) | | | | | |
|  | Control Symptoms | 2.68 (2.43 to 2.92) | 2.97 (2.74 to 3.20) | –0.30 (–0.63 to 0.04) | .08 |
|  | Control Symptoms (2 outliers removed) | 2.75 (2.54 to 2.96) | 3.03 (2.83 to 3.23) | –0.28 (–0.57 to 0.01) | .06 |
|  | Maintain Function | 2.31 (2.04 to 2.59) | 2.73 (2.47 to 2.99) | –0.41 (–0.79 to –0.36) | .03 |
|  | Maintain Function (1 outlier removed) | 2.39 (2.14 to 2.65) | 2.72 (2.48to 2.95) | –0.32 (-0.67 to 0.02) | .07 |
|  | Total | 2.52 (2.28 to 2.76) | 2.86 (2.64 to 3.08) | –0.34 (-0.67 to –0.02) | .04 |
|  | Total (1 outlier removed) | 2.60 (2.38 to 2.81) | 2.86 (2.66 to 3.06) | –0.26 (–0.55 to 0.03) | .08 |
|  | Total Plus | 2.45 (2.22 to 2.68) | 2.83 (2.62 to 3.05) | –0.38 (-0.70 to –0.07) | .02 |
|  | Total Plus (1 outlier removed) | 2.53 (2.32 to 2.74) | 2.83 (2.64 to 3.03) | –0.30 (-0.59 to –0.02) | .04 |
| Morisky Medication Adherence Scale | | 6.75 (6.34 to 7.16) | 7.05 (6.72 to 7.38) | –0.30 (-0.83 to 0.23) | .27 |

^a^The unadjusted model includes baseline scores as a covariate except for Morisky Medication Adherence Scale, which was not measured at baseline.

^b^In the Txt2Prevent group, 31 participants were analyzed for the EQ-5D-5L questionnaire. Thirty-two participants were analyzed for the remaining outcomes (excluding those with outliers removed). For the usual care group, 35 participants were analyzed for the heiQ: Health directed activity. Thirty-six participants were analyzed for the remaining outcomes (excluding those with outliers removed). For the two outliers for the CSE Control Symptoms domain, one was from the Txt2Prevent group and one was from the usual care group. The one outlier for the CSE Maintain Function, Total, and Total Plus was from the Txt2Prevent group.
