# Supplementary material for: The Use of SMS Text Messaging to Improve the Hospital-to-Community Transition in Patients With Acute Coronary Syndrome (Txt2Prevent): Results From a Pilot Randomized Controlled Trial
Source: JMIR Mhealth Uhealth. 2021 May 14;9(5):e24530. doi: 10.2196/24530 (PMC8164115; doi:10.2196/24530)
Supplement: Multimedia Appendix 3 [file mhealth_v9i5e24530_app3.docx]

Multimedia Appendix 3. 60-day follow-up medication prescriptions, by group.

|  | Txt2Prevent  (n=31/32)^a^, n(%) | Usual care  (n=35/36)^a^, n(%) | *P*-value |
| --- | --- | --- | --- |
| Acetylsalicylic acid | 29 (94) | 31 (89) | .68 |
| Ticagrelor or clopidogrel | 25 (81) | 33 (92) | .28 |
| Statin | 32 (100) | 32 (91) | .24 |
| Beta blocker | 28 (90) | 31 (89) | 1.00 |
| Angiotensin-converting-enzyme inhibitor or angiotensin II receptor blocker | 27 (87) | 33 (94) | .41 |

^a^In the Txt2Prevent group, 32 participants were analyzed for statins. Thirty-one were analyzed for the remaining medication categories. In the usual care group, 36 were analyzed for Ticagrelor/Clopidogrel. Thirty-five were analyzed for the remaining medication categories.
