# Supplementary material for: The Use of SMS Text Messaging to Improve the Hospital-to-Community Transition in Patients With Acute Coronary Syndrome (Txt2Prevent): Results From a Pilot Randomized Controlled Trial
Source: JMIR Mhealth Uhealth. 2021 May 14;9(5):e24530. doi: 10.2196/24530 (PMC8164115; doi:10.2196/24530)
Supplement: Multimedia Appendix 4 [file mhealth_v9i5e24530_app4.docx]

Multimedia Appendix 4. Physician visits and cardiac rehabilitation enrolment within 60-days, by group.

|  | Txt2Prevent (n=32), n (%) | Usual care (n=35/36)^b^, n (%) | Unadjusted relative risk (95% CI) | *P*-value | Adjusted relative risk (95% CI)^a^ | *P*-value |
| --- | --- | --- | --- | --- | --- | --- |
| Visited a family physician | 29 (91) | 34 (94) | 0.96 (0.84 to 1.10) | .55 | 0.93 (0.82 to 1.04) | .21 |
| Visited a cardiologist | 15 (47) | 26 (72) | 0.65 (0.43 to 0.99) | .04 | 0.73 (0.48 to 1.10) | .13 |
| Joined a cardiac rehabilitation program | 7 (22) | 10 (29) | 0.77 (0.33 to 1.77) | .53 | 0.90 (0.43 to 1.89) | .78 |
| ^a^The adjusted model includes age, sex, geographic region and income.  ^b^For the usual care group, 35 participants were analyzed for whether they joined a cardiac rehabilitation program. Thirty-six participants were analyzed for whether they had visited family physician or cardiologist. | | | | | | |
